# Myxoid Lipoblastoma with New Fusion Transcript *CHCHD7::PLAG1* in an 18-Month-Old Girl Diagnosed by Target RNA Sequencing: A Case Report

**DOI:** 10.3390/ijms27104312

**Published:** 2026-05-12

**Authors:** Danijela Cvetković, Marina Gazdić Janković, Marina Miletić Kovačević, Amra Ramović Hamzagić, Irena Urošević, Vesna Rosić, Biljana Ljujić

**Affiliations:** 1 Department of Genetics, Faculty of Medical Sciences, University of Kragujevac, 34000 Kragujevac, Serbiamarinagazdic87@gmail.com (M.G.J.); bljujic74@gmail.com (B.L.); 2 Department of Histology and Embryology, Faculty of Medical Sciences, University of Kragujevac, 34000 Kragujevac, Serbia; vecarosic@gmail.com; 3 Center for Genetics and Molecular Diagnostics, Polyclinic Ramovic, 36300 Novi Pazar, Serbia; ramovicamra@gmail.com; 4 Faculty of Medical Sciences, University of Kragujevac, 34000 Kragujevac, Serbia; ire996na@gmail.com

**Keywords:** *PLAG1*, lipoblastoma, toddler, chromosomal rearrangement, RNA sequence

## Abstract

Lipoblastomas are rare, benign tumors arising from embryonic white fatty precursor cells that continue to proliferate in the postnatal period. We present a case of a minimally differentiated lipoblastoma with myxoid features. Our patient was an 18-month-old female with a painless solid tumefaction in the middle third of the right leg. Histopathologically, the nodular tumor mass consisted of lipoblasts embedded in a myxoid stroma. Immunohistochemistry showed strong diffuse positivity for S100, CD34, CD56, NSE and rare Ki67+ cells. FOXO1 polyploidy was detected in 30% of cells by FISH. Using target RNA sequencing, we detected a *CHCHD7::PLAG1* fusion gene showing that the first exons of *CHCHD7* were fused to either exon 2 or exon 3 of *PLAG1*. Our case demonstrates that due to the histomorphologic overlaps, the molecular diagnostics can be essential for the confirmation of the diagnosis of lipoblastoma.

## 1. Introduction

Lipoblastomas are rare, benign soft-tissue tumors arising from embryonic white fat precursor cells that continue to proliferate mainly in the postnatal period [1]. This tumor primarily affects children younger than 3 years of age (75–90%), but can also occur in older children and very rarely in adulthood [2]. Lipoblastoma is a well-encapsulated mass and no malignant transformation and metastasis has been documented to date. The long-term prognosis for lipoblastoma is excellent. This tumor is usually located in the subcutaneous tissue of the trunk and extremities; however, other less common tumor sites such as the head and neck, mediastinum, mesentery, retroperitoneum, inguinoscrotal or labial region have also been reported [1]. Thus, depending on tumor location, a soft palpable mass causes a broad range of clinical features. Macroscopically, lipoblastoma is a lobulated, soft, and encapsulated mass of yellow or creamy white color. Histopathologically, lipoblastoma is characterized by different stages of maturation ranging from stellate or spindled mesenchymal cells, mono- or multivacuolated lipoblasts, to mature adipocytes. Lipoblasts are cells that contain a hyperchromatic nucleus and a lipid-rich cytoplasm, which may be mono- or multivacuolar. The nucleus may be located eccentrically if a single large fat droplet is present, or centrally with smaller indentations formed by the interaction of multiple small lipid droplets [3]. Fat cells are separated by well-vascularized fibrovascular septa and areas of a myxoid matrix with primitive stellate or spindled mesenchymal cells and plexiform vascular network [4,5]. Lipoblastoma in a late stage may show fibrolipomatous changes without the presence of lipoblasts [6]. According to histopathological and clinical features, there are two types of lipoblastoma: circumscribed and diffuse lipoblastoma. The circumscribed lipoblastoma is more common; it is encapsulated and most often found in the subcutaneous tissue of the limbs. Diffuse lipoblastoma, more commonly known as lipoblastomatosis, occurs less frequently and is characterized by infiltrative and diffuse growth, deep localization and absence of capsule [7]. The goal of treatment is complete surgical resection of the tumor; however, this is difficult to achieve in lipoblastomatosis. In these cases, especially in newborns, conservative treatment is used [8,9]. To date, no spontaneous regression of lipoblastoma has been reported, and recurrence rates range from 14% to 25% when incompletely excised.

Gene fusions based on chromosomal rearrangements are the main genetic alteration in lipoblastoma and have an important role in tumorigenesis [10]. Structural alteration of 8q11–q13 leads to a rearrangement of the pleomorphic adenoma gene 1 (*PLAG1*). *PLAG1* was first described in pleomorphic adenomas of the salivary glands and belongs to the PLAG family of transcription factors along with *PLAG*-like 1 and *PLAG*-like 2 genes [11]. *PLAG1* oncogene encodes a zinc finger transcription factor, which is broadly expressed during fetal development and only at very low levels postnatally [12]. *PLAG1* transcriptional up-regulation in lipoblastoma is due to the gene rearrangements bringing the *PLAG1* gene under the transcriptional control of a more active promoter (promotor swapping) [13]. In particular, exon 2 or 3 of *PLAG1* fused with exon 1 of the N-terminal side of the partner genes causes increased transcription of the fusion gene [14]. *PLAG1* rearrangements have been detected in 60–70% of lipoblastomas by classic cytogenetic analysis, fluorescence in situ hybridization (FISH), and targeted RNA sequencing approaches. The most commonly described fusion partners are the *HAS2* (8q24.1), *COL1A2* (7q21.3), *COL3A1*, *RAB2A*, *RAD51L1* (aka RAD51B), *BOC*, *ZEB2*, *DDX6*, *KLF10*, *KANSLIL*, *HMGA2*, *RUNX1T1*, *SRSF3*, *PCMTD1*, *YWHAZ*, *CTDSP2*, *PPP2R2A*, *HNRNPC*, *EEF1A1*, *PI15* (8q21.13), *DLEU2* and *VCAN* genes, while the *CHCHD7* fusion has been mostly reported as a recurrent event in salivary gland pleomorphic adenoma but very rarely in lipoblastomas with different exon splice site (fusion) [6,15,16]. Here, we describe the diagnostic workup of a pediatric patient with a lipoblastoma, and compare these findings with other reports of this rare tumor. Our case demonstrates that molecular analyses can play an essential role in making unequivocal diagnosis of a myxoid lipoblastomas in infants without typical histological characteristics. To the best of our knowledge, this is the first case of lipoblastoma due to break points in chr8:57124396-chr8:57083748 and chr8:57124396-chr8:57092072 region 8.

## 2. Results

### 2.1. Clinical and Histopathological Features

An 18-month-old female had a painless solid lesion in the middle third of the right leg. Echosonographically, in the anterior part of the right leg, an oval hyperechoic change (dominantly central hyperechoic) was detected at about 6.3 mm from the skin level, measuring about 12 × 30 × 16 mm, which was at the level of the associated muscle. The hyperechoic zone did not involve bone. Radiographic analyses confirmed intact bone. Laboratory analysis showed resulting values within the normal range.

**Figure 1:**
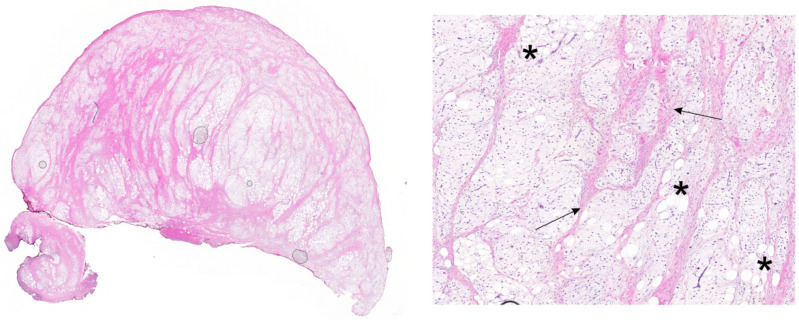
Hematoxylin and eosin (H&E) staining. H&E showed lobules of mature fat cells and lipoblasts separated by fibrous septa (black arrow). Mature adipocytes have small eccentrically positioned nuclei that are compressed (black asterisk). The tumor is pseudo-encapsulated with a nodular growth pattern. The lipoblastic cells are embedded in a myxoid stroma which in places has a chondroid appearance.

The tumor was completely removed and sent for histopathological investigation. The operative and immediate postoperative course were uneventful. Pathological examination showed a white-yellowish lobulated and encapsulated soft tissue lesion histopathologically comprised of spindle-and stelletate-shaped prelipoblasts and lipoblasts in different stages of maturation separated by fibrous vascularization septa. Myxoid areas were present which had a partially chondroid appearance (Figure 1). The results of the histopathological analysis raised suspicion of a myxoid lipoblastoma.

An additional challenge in establishing the correct diagnosis was the fact that the child had a family history of osteosarcoma and other malignancies, which increased the genetic burden.

Immunohistochemical analysis of the tumor showed strong diffuse positivity for S100 (+++), CD34 (++), CD56 (+++), NSE (+++) and rare Ki67+ positive cells (<1%). Immunohistochemical staining for myogenin, Myo D1, Rb, LCA, SOX10, alpha SMA and caldesmon were negative.

### 2.2. Cytogenetic and Molecular Features

Fluorescence in situ hybridization (FISH) represents a valuable method for detecting gene rearrangements and amplifications. This diagnostic approach provides a reliable and reproducible assay that aids in the differential diagnosis of various neoplasms and serves as a useful complement to histological and immunohistochemical analyses [17]. In order to exclude myxoid liposarcoma and PMMTI (primitive myxoid mesenchymal tumor of infancy), additional molecular testing was performed. In this context, FOXO1 FISH analysis demonstrated no rearrangement; instead, FOXO1 polyploidy was detected in 30% of cells. A *CHCHD7::PLAG1* was identified using the NGS somatic tumor panel (TUM01) and RNA sequencing resulting from a cryptic intrachromosomal rearrangement at 8q12.

The focal somatic copy number alterations and structural alterations detected in the tumor sample are presented in Supplementary Figure S1. Both *PLAG1* fusions identified in this study involved the 5′ untranslated regions of both PLAG1 and a fusion partner gene. In *CHCHD7*-*PLAG1*, *CHCHD7* (NM_001011671.3) exon 1 fused to *PLAG1* (NM_002655.3) exon 3 (Supplementary Figure S1A), and *CHCHD7* (NM_001011671.3) exon 1 fused to *PLAG1* (NM_002655.3) exon 2 (Supplementary Figure S1B). Consequently, all fusions resulted in having the entire *PLAG1* coding sequence, which begins in exon 4, placed under the transcriptional control of promoter regions of fusion partner genes (promoter swap).

Based on morphology and fusion gene analysis, the diagnosis of a lipoblastoma was made.

Our sequencing data do not provide evidence for the presence of potentially relevant copy number alterations of large genomic segments. There is no evidence for the presence of homozygous deletions or strong amplifications of single therapeutically relevant genes.

## 3. Discussion

Lipoblastoma commonly presents as a painless subcutaneous soft tissue mass; however, the differential diagnosis is broad and includes a range of benign and malignant entities. Lipoblastoma and lipoblastomatosis, along with lipomatosis and lipofibromatosis, represent benign adipose tissue tumors of pediatric patients, whereas liposarcomas are malignant neoplasms that are exceptionally rare in the pediatric population with myxoid liposarcoma being the most common [18,19] and the main differential diagnosis of lipoblastoma [15]. Although adipocytic tumors account for only about 6% of soft tissue tumors in children, approximately 95% of them are benign. Histologically, lipoblastoma is characterized by a lobulated architecture and is composed of prelipoblasts, lipoblasts and adipocytes separated by septa, without pleomorphism [1]. The extracellular matrix may be myxoid, which can resemble myxoid liposarcoma. Lipoblastoma most commonly occurs in the trunk of children under the age of three and follows a benign clinical course [2]. In contrast, myxoid liposarcoma is significantly less common in this age group and usually occurs in the extremities in patients aged 30–60 years. Histomorphologically, these lesions are also composed of lobules of prelipoblasts, lipoblasts and adipocytes with a typical condensation of primitive cells at the periphery of the lobules. There is a typical chicken-wire vasculature not seen in lipoblastoma [15,19].

The diagnostic procedure to identify lipoblastoma includes clinical and histopathological features, and molecular approaches due to the histomorphologic overlap, while molecular diagnostics with identification of *PLAG1*- rearrangement can be essential for confirmation of lipoblastoma, because it has its own characteristic genetic alterations.

Here, we present a case of a toddler with amyxoid neoplasm with features of a primitive looking lipoblastoma. The vast majority of lipoblastomas are characterized by rearrangement of the *PLAG1* gene, leading to *PLAG1* overexpression and tumorigenesis [20]. Using target RNA sequencing, we detected a fusion gene, *CHCHD7*::*PLAG1*. Sequence analysis showed that the first exons of *CHCHD7* were fused to either exon 2 or exon 3 of *PLAG1*. *PLAG1* has a genomic fusion breakpoint in intron 1, resulting in alternative splicing of exon 2. The start codon of *PLAG1* is located in exon 4, and the coding sequences of *PLAG1* are preserved in the *CHCHD7*::*PLAG1* fusion gene. Based on a review of the available literature, we came to the conclusion that the frequency of this tumor is relatively rare in the population of children. Furthermore, we reviewed the literature investigating fusion genes in lipoblastoma. In the period from 1990 to 2025, only 2 cases were published on PubMed, which supports the relevance of this topic.

When we added “potentially relevant fusion *CHCHD7::PLAG1* lipoblastoma” to the PubMed literature search, we found a total of one paper. Given that gene fusions or chromosomal rearrangements play an important role in tumorigenesis, this highlights the importance of our research.

Previous studies indicate that *PLAG1* activation induces IGF2 overexpression, promoting cellular proliferation and arresting adipocytic differentiation [11,12]. We hypothesize that the *CHCHD7* promoter drives ectopic *PLAG1* expression through a promoter-swap mechanism, similar to that observed in pleomorphic adenomas. Due to limited tissue availability, functional validation was not feasible; therefore, we classified this fusion as a candidate oncogenic event requiring further investigation into its role in lipoblastoma pathogenesis.

The identification of the *CHCHD7::PLAG1* fusion via RNA sequencing was decisive for the final diagnosis. Histopathological features, such as myxoid stroma and primitive lipoblasts, can mimic myxoid liposarcoma or PMMTI, while the immunohistochemical profile (S100, CD34, CD56) remained non-specific. Molecular confirmation of the *PLAG1* fusion, combined with the absence of pathogenic FOXO1 rearrangement, confirmed the diagnosis of lipoblastoma and prevented unnecessary aggressive therapy [6,13,14].

FOXO1 (13q14) rearrangement analysis is primarily used to exclude alveolar rhabdomyosarcoma [17]. In this case, FISH analysis detected FOXO1 polyploidy in ~30% of nuclei without gene break-apart signals. According to the literature, low-level FOXO1 polyploidy typically represents non-specific chromosomal duplication or technical artifact rather than a pathogenic driver. Consequently, this finding was interpreted as cytogenetically non-specific and not indicative of FOXO1-related oncogenesis.

## 4. Materials and Methods

### 4.1. Patient and Tumor Tissue Sample

Our patient was an 18-month-old female with no past medical, family or hereditary history relevant to the case, with a painless solid tumefaction (size of a grape) in the middle third of the right leg above the margo anterior tibiae.

### 4.2. Histopathological and Immunohistochemical Analyses

For histopathological analysis, the samples were fixed in 10% phosphate-buffered formalin, sectioned at 5 μm and stained with hematoxylin and eosin (H&E) stain (Sigma–Aldrich, St. Louis, MO, USA) for light microscopic examination. Immunohistochemistry was performed on deparaffinized, rehydrated sections obtained from a representative formalin-fixed, paraffin-embedded block using antibody-specific epitope retrieval techniques with the Dako Envision (Dako, Carpinteria, CA, USA) automated system for detection of the following primary antigens: vimentin, desmin, CD34, Myogenin, MyoD1, Rb, LCA, SOX10, alpha SMA, caldesmin, CD56, NSE, S100, and Ki67, according to the manufacturers’ instructions and standard protocols.

### 4.3. Fluorescence In Situ Hybridization

Interphase fluorescence in situ hybridization (FISH) for FOXO1 rearrangement was performed on FFPE tumor tissues using a locus specific dual-color break-apart FOXO1 (13q14.11) probe set according to the manufacturers’ instructions and standard protocols.

### 4.4. Next-Generation Sequencing and RNA-Sequencing

Tumor panel TUM01 was used in somatic molecular genetic analysis of a tumor tissue sample in order to evaluate somatic variants of potential clinical relevance. RNA fusions panel analysis (STR) was employed to identify gene fusions. The isolation of DNA and RNA from tumor in FFPE as well as normal DNA from normal tissue (EDTA blood) was performed. The tumor material was assessed by a pathology specialist. NGS-laboratory DNA: Protein-coding regions, as well as flanking intronic regions and additional disease-relevant non-coding regions, were enriched using in-solution hybridization technology, and were sequenced using the Illumina NovaSeq 6000/NovaSeq X Plus system at CeGaT GmbH, Tübingen, Germany.

NGS-laboratory RNA: RNA from tumor tissue was sequenced. Fusion transcripts were enriched using in-solution hybridization technology. For fusion transcripts with known breakpoints, breakpoint spanning probes were used. For genes with unknown breakpoints or a large number of possible fusion partners, the coding sequence was used for enrichment. Sequencing was performed on Illumina NovaSeq 6000/NovaSeq X Plus systems, while Illumina bcl2fastq2 was used to demultiplex sequencing reads at CeGaT GmbH, Tübingen, Germany.

Genetic data evaluation DNA: Only variants (SNVs/small indels) with a novel allele frequency (NAF) of 5% in the tumor sample within the coding regions and their adjacent intronic regions (−/+ 8 base pairs) were evaluated. A list of all the variants with an allele frequency of 5% considered in the genetic data evaluation can be requested at any time. The clinical interpretation of variants is based on different external and internal databases and on information from the scientific literature. The sensitivity of the test is dependent on the tumor content of the analyzed material, the sample quality, and the sequencing depth. In this case, 99.03% of the targeted regions were covered by a minimum of 70 high-quality sequencing reads per base. The diagnostic tumor content (expert estimate) was 60%. A theoretical sensitivity of >99% can be obtained for variants with a NAF 30% when a coverage of 35 reads per base is achieved. Variants are named according to the HGVS recommendations without any information regarding the cis or trans configuration.

Genetic data evaluation RNA: The sensitivity of the test is dependent on the tumor content of the analyzed material, the sample quality, and the amount of transcripts sequenced. In this case, an amount of 19.01 gigabases RNA was sequenced. Therefore, this analysis is appropriate to detect structural variants at the RNA level.

## 5. Conclusions

Recognition of this rare tumor is important because lipoblastoma can be misdiagnosed leading to overtreatment. Careful integration of clinical presentation, histopathological, immunohistochemical and molecular analysis facilitates the diagnosis of this rare entity in children. Targeted RNA-sequencing technology to demonstrate fusion transcripts affecting *PLAG1* are very useful as a diagnostic tool for the accurate diagnosis of lipoblastoma.

## Data Availability

The data presented in this study are available on request from the corresponding author.

## Supplementary Materials

The following supporting information can be downloaded at: https://www.mdpi.com/article/10.3390/ijms27104312/s1.
