# Supplementary material for: Myxoid Lipoblastoma with New Fusion Transcript CHCHD7::PLAG1 in an 18-Month-Old Girl Diagnosed by Target RNA Sequencing: A Case Report
Source: Int J Mol Sci. 2026 May 12;27(10):4312. doi: 10.3390/ijms27104312 (PMC13207982; doi:10.3390/ijms27104312)
Supplement: Supplementary file 1 [file ijms-27-04312-s001.zip › ijms-4306143-supplementary.pdf]

Supplementary Data: “Myxoid lipoblastoma with new fusion transcript *CHCHD7*::*PLAG1* in an 18-month-old girl diagnosed by target RNA sequencing: a case report”

Figure S1

A

Gene	Functional category	Transcript-ID
CHCHD7-PLAG1 chr8:57124396-chr8:57083748	fusion CHCHD7 5’UTR (exon 1), PLAG1 5’UTR (exon 3)	NM_001011671.3, NM_002655.3

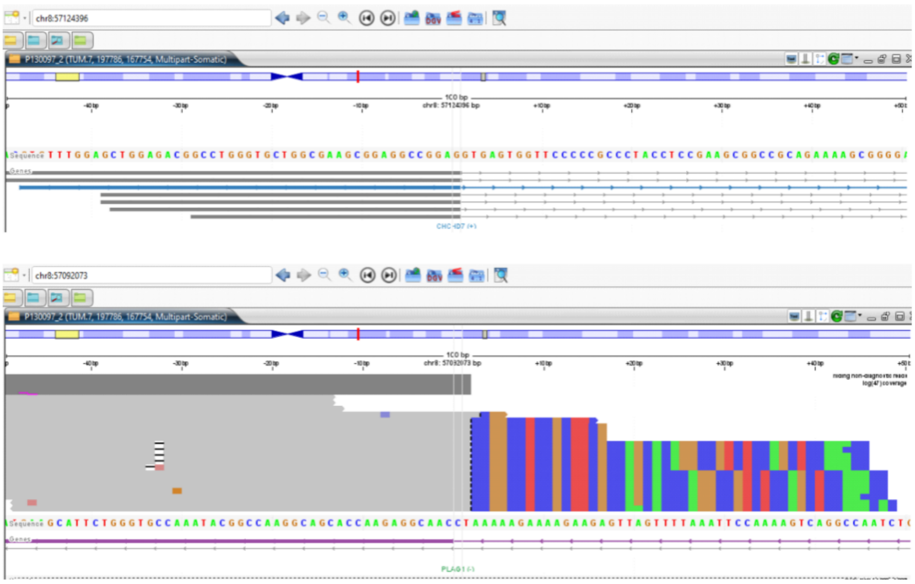

B

Gene	Functional category	Transcript-ID
CHCHD7-PLAG1 chr8:57124396-chr8:57092072	fusion CHCHD7 5’UTR (exon 1), PLAG1 5’UTR (exon 2)	NM_001011671.3, NM_002655.3

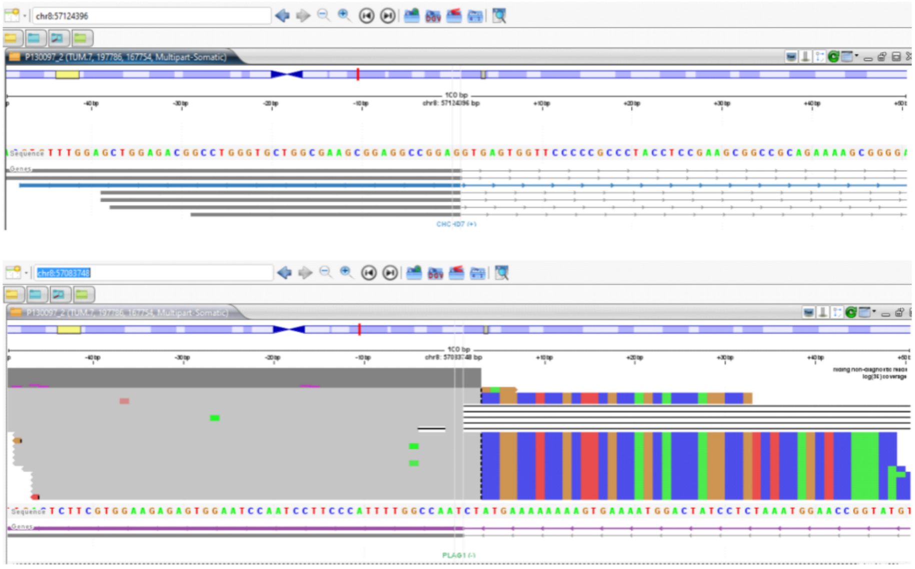

Figure S1. Focal somatic copy number alterations and structural alterations detected in the tumor sample. (A) CHCHD7-PLAG1, CHCHD7 (NM_001011671.3) exon 1 fused to PLAG1

(NM_002655.3) exon 3. (B) CHCHD7 (NM_001011671.3) exon 1 fused to PLAG1 (NM_002655.3) exon 2.
